# Adherence to monitoring iron indices at the initiation of erythropoiesis-stimulating agents or hypoxia-inducible factor prolyl hydroxylase inhibitors

**DOI:** 10.1007/s10157-025-02761-1

**Published:** 2025-09-16

**Authors:** Yoshihisa Miyamoto, Akira Okada, Yusuke Sasabuchi, Masaomi Nangaku, Hideo Yasunaga

**Affiliations:** 1 Department of Real-World Evidence, The University of Tokyo, 7-3-1 Hongo, Bunkyo-Ku, Tokyo, 113-0033 Japan; 2 Department of Prevention of Diabetes and Lifestyle-Related Diseases, Graduate School of Medicine, The University of Tokyo, 7-3-1 Hongo, Bunkyo-ku, Tokyo, 113-0033 Japan; 3 Division of Nephrology and Endocrinology, The University of Tokyo, 7-3-1 Hongo, Bunkyo-Ku, Tokyo, 113-0033 Japan; 4 Department of Clinical Epidemiology and Health Economics, School of Public Health, The University of Tokyo, 7-3-1 Hongo, Bunkyo-Ku, Tokyo, 113-0033 Japan

**Keywords:** Erythropoiesis-stimulating agents, Hypoxia-inducible factor prolyl hydroxylase inhibitors, Adherence

## Abstract

**Background:**

Hypoxia-inducible factor prolyl hydroxylase (HIF-PH) inhibitors have been used for the treatment of anemia in patients with chronic kidney disease not receiving dialysis since 2020. In September 2020, the Japanese Society of Nephrology published recommendations for the appropriate use of HIF-PH inhibitors, which emphasized monitoring iron indices. However, real-world adherence to these recommendations remains unclear.

**Methods:**

We retrieved the data of new users of erythropoietin-stimulating agents (ESAs) or HIF-PH inhibitors from a large Japanese claims database (DeSC, Tokyo, Japan) between 2018 and 2022. Adherence to iron testing before and after the treatments was analyzed using modified Poisson regression and Cox models. Facility-level variations were assessed via mixed-effects models.

**Results:**

We identified 105,346 patients who had a new prescription of ESAs (*n* = 86,263) or HIF-PH inhibitors (*n* = 19,083) and did not have kidney failure with replacement therapy. The proportion of HIF-PH inhibitor use increased from 3.6% in 2020 to 42.7% in 2022. During the study period, testing frequency for serum iron, serum TIBC or UIBC, and ferritin ranged from 57.2–59.8%, 39.2–42.8%, and 50.6–52.6%, respectively. Multivariate analysis showed that adherence to testing was significantly higher in university hospitals, Diagnosis Procedure Combination-affiliated DPC hospitals, and non-DPC hospitals compared with clinics. A similar tendency was observed in testing after the index date.

**Conclusions:**

The type of facility was the primary determinant of adherence to the recommendation for iron indices testing before the initiation of ESAs or HIF-PH inhibitors. Targeted educational interventions in low-adherence settings may help improve adherence rates and optimize patient care.

**Supplementary Information:**

The online version contains supplementary material available at 10.1007/s10157-025-02761-1.

## Introduction

Anemia in chronic kidney disease (CKD) represents a multifactorial complication, whose incidence increases with the decline in kidney function [1–3]. Anemia in CKD is not only associated with symptoms such as fatigue, but also contributes to an elevated risk of cardiovascular events, hospitalization, and mortality [4]. While decreased erythropoietin production is the hallmark pathophysiology of anemia in CKD, iron deficiency is a critical modifiable contributor, affecting both erythropoietic response and treatment outcomes [5–7]. Iron indices should be measured to assess iron status in patients with CKD, and iron repletion should be considered when the patient has iron deficiency.

Erythropoiesis-stimulating agents (ESAs) and hypoxia-inducible factor prolyl hydroxylase (HIF-PH) inhibitors constitute key treatments for anemia in CKD [8]. While ESAs have long been used to stimulate red blood cell production, HIF-PH inhibitors have a novel mechanism of action that entails enhancing endogenous erythropoietin production and improving iron metabolism. Both therapies emphasize the importance of addressing iron deficiency during treatment. The package inserts of ESAs or HIF-PH inhibitors warn that iron supplementation is essential in the event of iron deficiency. Recently accrued data suggest that the depletion in iron levels after the initiation of HIF-PH inhibitors arising from the increased availability of iron for erythropoiesis may be associated with a higher risk of thrombotic events [9, 10]. Thus, some expert societies have advocated testing for iron indices, followed by iron repletion, if needed [11–14]. Notably, the Japanese Society of Nephrology published recommendations on the appropriate use of HIF-PH inhibitors in 2020.

Although laboratory monitoring is generally deemed essential for optimizing medication use, the current testing practices for various medications are reportedly suboptimal [15–17]. Meanwhile, there is a paucity of data on pre-treatment monitoring for newer agents, including HIF-PH inhibitors. Therefore, we conducted a retrospective study to assess the frequency of recommendation-adherent testing before and after initiating ESAs or HIF-PH inhibitors. We also aimed to explore the factors associated with recommendation-adherent testing, including provider- and patient-related characteristics.

## Materials and methods

### Data source

This study analyzed data extracted from the DeSC administrative claims database (DeSC Healthcare Inc., Tokyo, Japan). The DeSC database is a repository of administrative claims data from clinics, hospitals, and pharmacies. The population contained within the DeSC database is representative of the entire population of Japan. The database includes data on medical institutions, diagnostic codes based on the International Statistical Classification of Diseases and Related Health Problems, 10th Revision (ICD-10), detailed medical procedures, and drug dispensations. The details are described elsewhere [18]. This study was approved by the Institutional Review Board of The University of Tokyo [approval number: 2021010NI (April 23, 2021)] and adhered to the Declaration of Helsinki. The requirement for patient-informed consent was waived because all the data were de-identified.

### Study participants and study period

Patients who were given at least one prescription of ESAs or HIF-PH inhibitors between 2018 and 2022 were included. The following ESAs are available for the treatment of anemia in CKD: epoetin alfa, epoetin beta, darbepoetin alfa, epoetin beta pegol, and their biosimilars. In Japan, five HIF‐PH inhibitors, including roxadustat, vadadustat, daprodustat, enarodustat, and molidustat, have been approved for patients with anemia in CKD, including those not receiving dialysis [19].

We defined the index date as the first prescription date of ESAs or HIF-PH inhibitors. We excluded (1) patients who had a baseline period of less than 6 months before the first prescription of ESAs or HIF-PH inhibitors (*n* = 56,503), (2) those who had undergone autologous blood donation within 90 days before the index date (*n* = 7,381) because some ESA formulations are covered by insurance when used for autologous blood storage, (3) those who were diagnosed with myelodysplastic syndrome within 3 months before the index date (*n* = 2,922), (4) those who had undergone renal replacement therapy within 90 days before the index date (*n* = 15,969), and (5) those who received both ESAs and HIF-PH inhibitors on the same date (*n* = 25).

### Outcomes and covariates

The primary outcome was whether laboratory testing for monitoring iron indices was performed within 90 days before and after the index date. The iron indices included serum iron, total or unsaturated iron-binding capacity (TIBC or UIBC), and ferritin. We extracted all test results before the administration of ESA or HIF-PH inhibitors, irrespective of the institution at which they were conducted. This is because it is possible for the physician who initiated ESA or HIF-PH inhibitors to check the laboratory results obtained at another institution. We adopted the approach where the physicians who initiated ESA or HIF-PH inhibitors were responsible for checking the results, irrespective of where the laboratory tests were performed. This approach conforms with the primary care physician model and dual-attending physician system.

The covariates included the year of prescription (from 2018 to 2022), drug type (ESA or HIF-PH inhibitor), patient’s sex, insurance type (non-employee, employee, and late elderly), and type of facility [clinic, university hospital, Diagnosis Procedure Combination (DPC)-hospital besides a university hospital, and non-DPC hospital]. A DPC hospital is a medical facility that operates under a per diem, prospective payment system, wherein the hospital is reimbursed at a fixed daily rate determined by the patient’s primary diagnosis and treatment. DPC hospitals are likely to be larger than non-DPC hospitals. We also examined complete blood count (CBC) testing within 90 days before the index date to determine whether the differences in adherence could be attributed to variations in the frequency of visits or routine testing at healthcare facilities.

### Statistical analysis

To explore the relevant determinants of adherence to testing, we considered a hierarchical modified Poisson regression model to estimate the prevalence ratio between the groups of relevant factors [20]. The model included year of prescription (from 2018 to 2022), drug type (ESA or HIF-PH inhibitor), patient’s sex, insurance type, and type of facility. We treated facilities as a random intercept to account for clustering within treatment facilities [21]. We estimated the median prevalence ratio to quantify residual facility-level variations unexplained by the covariates in the model. A median prevalence ratio of 1.0 indicates that the prevalence of adherence is equivalent across facilities; a larger median prevalence ratio indicates greater variations among the facilities [22]. Multilevel models were constructed using the glmmTMB package.

To assess adherence to testing following the initiation of ESAs or HIF-PH inhibitors, we conducted a time-to-event analysis to evaluate the factors relevant to iron index monitoring. The factors were the same as those included in the modified Poisson regression. We considered multivariable Cox proportional hazards models with a log-normal frailty term to account for unobserved heterogeneity across facilities [23]. The observation period commenced from the index date (first prescription of ESAs or HIF-PH inhibitors) and continued until the earliest occurrence of the first measurement of iron indices, death, disenrollment from the insurance plan, or 90 days after the index date. We presented the hazard ratios from the Cox models and the cumulative incidences of the measurements at 90 days after the index date. We also presented the median hazard ratio [24]. Analyses were conducted using R4.3.3 (R Core Team, R Foundation for Statistical Computing, Vienna, Austria).

## Results

The study included 105,346 patients who received a prescription of either ESAs (*n* = 86,263) or HIF-PH inhibitors (*n* = 19,083) across 9,043 different facilities. The patients’ demographics are presented in Table 1.

**Table 1:** Baseline characteristics of new users of ESAs and HIF-PH inhibitors

Characteristics	ESAs	HIF-PH inhibitors
Number of participants	86,263	19,083
Age, mean (SD)	82.52 (9.72)	84.77 (7.94)
Year of prescription, *n* (%)		
2018	9418 (10.9)	0 (0.0)
2019	19,530 (22.6)	0 (0.0)
2020	20,571 (23.8)	774 (4.1)
2021	20,676 (24.0)	6313 (33.1)
2022	16,068 (18.6)	11,996 (62.9)
Sex, Female, *n* (%)	42,052 (48.7)	10,433 (54.7)
Type of insurance, *n* (%)		
Employee	506 (0.6)	46 (0.2)
Late elderly	74,933 (86.9)	17,679 (92.6)
Non-employee	10,824 (12.5)	1358 (7.1)
Type of practice (%)		
Clinic	27,536 (31.9)	10,281 (53.9)
DPC hospital	38,713 (44.9)	5644 (29.6)
Non-DPC hospital	14,990 (17.4)	2577 (13.5)
University hospital	5024 (5.8)	581 (3.0)
Comorbidities		
Chronic kidney disease, *n* (%)	49,991 (58.0)	9370 (49.1)
Stroke, *n* (%)	10,462 (12.1)	2128 (11.2)
Congestive heart failure, *n* (%)	52,326 (60.7)	11,779 (61.7)
Myocardial infarction, *n* (%)	6880 (8.0)	1410 (7.4)
Pharmacological treatments		
Oral iron, *n* (%)	19,274 (22.3)	5603 (29.4)
Intravenous iron, *n* (%)	5489 (6.4)	621 (3.3)
Anti-hyperglycemic drugs, *n* (%)	29,789 (34.5)	5921 (31.0)
Antihyperlipidemic drugs, *n* (%)	37,092 (43.0)	8480 (44.4)
Anti-hypertensive drugs, *n* (%)	79,295 (91.9)	17,567 (92.1)
Vitamin D or derivatives, *n* (%)	14,900 (17.3)	3303 (17.3)
Iron-containing phosphate binders, *n* (%)	511 (0.6)	131 (0.7)
Non-iron-containing phosphate binders, *n* (%)	1156 (1.3)	62 (0.3)

*DPC* Diagnosis Procedure Combination, *ESAs* erythropoiesis-stimulating agents, *HIF-PH* hypoxia-inducible factor-prolyl hydroxylase

During the study period, testing prevalence ranged from 57.2% to 59.8% for serum iron, 39.2–42.8% for TIBC or UIBC and 50.6–52.6% for ferritin (Table 2). In comparison, testing for CBC remained consistently high, ranging from 98.2 to 98.6%.

**Table 2:** Prevalence and prevalence ratios of the relevant tests within 90 days before the first prescription of ESAs and HIF-PH inhibitors

Characteristics	*N*	Prevalence of testing (TIBC or UIBC)	PR for testing TIBC or UIBC)	Prevalence of testing (serum iron)	PR for testing (serum iron)	Prevalence of testing (ferritin)	PR for testing (ferritin)	Prevalence of testing (CBC)	PR for testing (CBC)
Year									
2018	9,418	39.2 [38.2, 40.2]	1 (ref)	57.2 [56.2, 58.2]	1 (ref)	50.6 [49.6, 51.6]	1 (ref)	98.4 [98.2, 98.6]	1 (ref)
2019	19,530	39.0 [38.4, 39.6]	1.02 [0.98, 1.06]	56.2 [55.4, 57.0]	0.99 [0.96, 1.02]	50.0 [49.2, 50.8]	1.01 [0.98, 1.05]	98.6 [98.4, 98.8]	1.00 [0.98, 1.03]
2020	21,345	39.4 [38.8, 40.0]	1.06 [1.01, 1.10]	57.2 [56.6, 57.8]	1.03 [0.99, 1.06]	51.2 [50.6, 51.8]	1.06 [1.02, 1.09]	98.6 [98.4, 98.8]	1.00 [0.98, 1.03]
2021	26,989	40.6 [40.0, 41.2]	1.11 [1.07, 1.16]	58.1 [57.5, 58.7]	1.06 [1.02, 1.09]	52.6 [52.0, 53.2]	1.11 [1.07, 1.15]	98.3 [98.1, 98.5]	1.00 [0.98, 1.03]
2022	28,064	42.8 [42.2, 43.4]	1.17 [1.12, 1.22]	59.8 [59.2, 60.4]	1.09 [1.06, 1.13]	55.3 [54.7, 55.9]	1.17 [1.13, 1.21]	98.2 [98.0, 98.4]	1.01 [0.98, 1.03]
Type of drug									
HIF-PH inhibitor	19,083	36.2 [35.6, 36.8]	0.97 [0.94, 1.00]	54.9 [54.1, 55.7]	0.96 [0.94, 0.99]	48.0 [47.2, 48.8]	0.95 [0.93, 0.98]	96.9 [96.7, 97.1]	0.98 [0.97, 1.00]
ESA	86,263	41.5 [41.1, 41.9]	1 (ref)	58.6 [58.2, 59.0]	1 (ref)	53.4 [53.0, 53.8]	1 (ref)	98.7 [98.7, 98.7]	1 (ref)
Sex									
Men	52,861	41.8 [41.4, 42.2]	1 (ref)	57.5 [57.1, 57.9]	1 (ref)	53.2 [52.8, 53.6]	1 (ref)	98.9 [98.9, 98.9]	1 (ref)
Women	52,485	39.3 [38.9, 39.7]	1.04 [1.02, 1.06]	58.4 [58.0, 58.8]	1.05 [1.03, 1.07]	51.6 [51.2, 52.0]	1.04 [1.03, 1.06]	97.9 [97.7, 98.1]	0.99 [0.98, 1.01]
Type of Insurance									
Late elderly	92,612	39.6 [39.2, 40.0]	1.01 [0.88, 1.16]	57.9 [57.5, 58.3]	1.07 [0.95, 1.20]	51.8 [51.4, 52.2]	1.04 [0.92, 1.18]	98.3 [98.3, 98.3]	1.02 [0.94, 1.11]
Non-employee	12,182	47.3 [46.3, 48.3]	1.02 [0.88, 1.17]	58.1 [57.3, 58.9]	1.03 [0.92, 1.16]	56.8 [56.0, 57.6]	1.03 [0.91, 1.17]	98.9 [98.7, 99.1]	1.02 [0.93, 1.11]
Employee	552	46.6 [42.5, 50.7]	1 (ref)	56.7 [52.6, 60.8]	1 (ref)	55.1 [51.0, 59.2]	1 (ref)	97.1 [95.7, 98.5]	1 (ref)
Type of facility									
University hospital	5,605	60.4 [59.0, 61.8]	2.62 [2.21, 3.11]	69.3 [68.1, 70.5]	1.39 [1.26, 1.54]	66.4 [65.2, 67.6]	1.82 [1.62, 2.04]	99.0 [98.8, 99.2]	1.02 [0.99, 1.05]
DPC hospital	44,357	51.9 [51.5, 52.3]	2.13 [1.99, 2.27]	64.0 [63.6, 64.4]	1.28 [1.23, 1.33]	63.9 [63.5, 64.3]	1.66 [1.59, 1.74]	99.7 [99.7, 99.7]	1.03 [1.01, 1.04]
Other hospital	17,567	36.6 [35.8, 37.4]	1.39 [1.31, 1.48]	57.1 [56.3, 57.9]	1.14 [1.10, 1.18]	47.5 [46.7, 48.3]	1.19 [1.14, 1.24]	98.4 [98.2, 98.6]	1.02 [1.00, 1.03]
Clinic	37,817	26.0 [25.6, 26.4]	1 (ref)	49.6 [49.0, 50.2]	1 (ref)	39.1 [38.5, 39.7]	1 (ref)	96.8 [96.6, 97.0]	1 (ref)

The prevalence and prevalence ratio are presented with the 95% confidence intervals

The prevalence of testing for the years 2018 through 2022 is presented in the rows corresponding to the respective year. The additional rows illustrate the prevalence of testing within the overall population, stratified by factors such as drug type or insurance type

Prevalence ratios were estimated using the hierarchical modified Poisson model. The model incorporated all covariates simultaneously

*CBC* complete blood count, *DPC* Diagnosis Procedure Combination, *ESAs* erythropoiesis-stimulating agents, *HIF-PH* hypoxia-inducible factor-prolyl hydroxylase, *PR* prevalence ratio, *TIBC or UIBC* total iron binding capacity or unsaturated iron binding capacity

Multivariable analysis showed that later year of prescription, female sex, and type of facility were associated with higher adherence to testing. Compared with clinics, adherence to testing was significantly higher in university hospitals, DPC hospitals, and non-DPC hospitals. The difference was marked in ferritin [adjusted prevalence ratio 1.82 (95% CI 1.62, 2.04) in university hospitals, 1.66 (95% CI 1.59, 1.74) in DPC hospitals] and TIBC or UIBC [adjusted prevalence ratio 2.62 (95% CI 2.21, 3.11) in university hospitals, 2.13 (95% CI 1.99, 2.27) in DPC hospitals], whereas the adjusted prevalence ratio was less notable in the measurement of iron [adjusted prevalence ratio 1.39 (95% CI 1.26, 1.54) in university hospitals, 1.28 (95% CI 1.23, 1.33) in DPC hospitals] (Table 2).

The median prevalence ratio for CBC testing was approximately 1, whereas the median prevalence ratios for testing of iron indices were 1.93 for TIBC or UIBC, 1.35 for iron, and 1.46 for ferritin.

As for testing after the index date, over the years, the cumulative incidence of testing for serum iron, serum TIBC or UIBC, and ferritin ranged from 44.4–48.7%, 22.9–33.5%, and 39.4–43.7%, respectively (Table 3). In contrast, testing for CBC remained consistently high during the study period, ranging from 93.1% to 94.5%. Similar to the prevalence ratio before the index date, the type of facility was strongly associated with the measurement of iron indices (Table 3). The median hazard ratios for iron index testing were 2.26 for TIBC or UIBC, 1.91 for iron, and 1.87 for ferritin, all of which were higher than the median hazard ratio of 1.45 for CBC testing.

**Table 3:** Cumulative incidences and hazard ratios of the relevant tests within 90 days after the first prescription of ESAs and HIF-PH inhibitors

Characteristics	CIF for testing (TIBC/UIBC)	HR for testing (TIBC/UIBC)	CIF for testing (serum iron)	HR for testing (serum iron)	CIF for testing (ferritin)	HR for testing (ferritin)	CIF for testing (CBC)	HR for testing (CBC)
Year								
2018	29.8 [28.9, 30.8]	1 (ref)	44.4 [43.4, 45.5]	1 (ref)	39.4 [38.4, 40.4]	1 (ref)	94.5 [94.0, 94.9]	1 (ref)
2019	29.8 [29.2, 30.5]	1.09 [1.04, 1.15]	44.7 [44.0, 45.3]	1.07 [1.03, 1.11]	38.1 [37.4, 38.7]	1.04 [1.00, 1.08]	94.4 [94.1, 94.7]	1.01 [0.98, 1.04]
2020	30.2 [29.6, 30.8]	1.15 [1.10, 1.21]	44.8 [44.1, 45.4]	1.10 [1.06, 1.14]	39.1 [38.5, 39.8]	1.11 [1.06, 1.15]	94.3 [94.0, 94.6]	1.02 [0.99, 1.05]
2021	32.5 [32.0, 33.1]	1.28 [1.23, 1.34]	47.1 [46.5, 47.7]	1.19 [1.15, 1.24]	41.9 [41.3, 42.5]	1.22 [1.17, 1.27]	93.7 [93.4, 94.0]	1.04 [1.01, 1.06]
2022	33.5 [33.0, 34.1]	1.32 [1.26, 1.39]	48.7 [48.2, 49.3]	1.22 [1.17, 1.27]	43.7 [43.1, 44.3]	1.27 [1.22, 1.32]	93.1 [92.8, 93.4]	1.02 [0.99, 1.04]
Type of drug								
HIF-PH inhibitor	30.4 [29.7, 31.1]	1.05 [1.01, 1.09]	47.2 [46.4, 47.9]	1.07 [1.04, 1.10]	40.8 [40.1, 41.5]	1.06 [1.03, 1.10]	91.3 [90.9, 91.7]	0.85 [0.83, 0.87]
ESA	31.8 [31.5, 32.2]	1 (ref)	46.2 [45.9, 46.5]	1 (ref)	40.9 [40.6, 41.2]	1 (ref)	94.4 [94.3, 94.6]	1 (ref)
Sex								
Men	34.4 [34.0, 34.8]	1 (ref)	47.8 [47.4, 48.3]	1 (ref)	43.5 [43.0, 43.9]	1 (ref)	95.3 [95.1, 95.5]	1 (ref)
Women	28.7 [28.3, 29.1]	0.93 [0.91, 0.95]	44.9 [44.5, 45.3]	0.98 [0.96, 1.00]	38.3 [37.9, 38.7]	0.95 [0.93, 0.97]	92.5 [92.2, 92.7]	0.94 [0.93, 0.95]
Type of Insurance								
Late elderly	30.0 [29.7, 30.3]	0.74 [0.64, 0.85]	45.5 [45.2, 45.9]	0.83 [0.73, 0.95]	39.5 [39.2, 39.8]	0.75 [0.66, 0.85]	93.4 [93.3, 93.6]	1.19 [1.08, 1.31]
Non-employee	43.4 [42.5, 44.3]	0.93 [0.80, 1.08]	52.3 [51.4, 53.2]	0.92 [0.81, 1.05]	50.8 [49.9, 51.7]	0.87 [0.77, 0.99]	97.1 [96.8, 97.4]	1.23 [1.12, 1.36]
Employee	45.4 [41.1, 49.5]	1 (ref)	56.5 [52.2, 60.6]	1 (ref)	55.8 [51.5, 59.9]	1 (ref)	95.3 [93.1, 96.8]	1 (ref)
Type of facility								
University hospital	50.5 [49.2, 51.8]	2.84 [2.32, 3.48]	60.1 [58.8, 61.3]	1.70 [1.44, 2.01]	56.5 [55.2, 57.8]	2.29 [1.95, 2.70]	97.5 [97.1, 97.9]	1.64 [1.48, 1.82]
DPC hospital	40.6 [40.1, 41.0]	2.12 [2.00, 2.25]	52.2 [51.8, 52.7]	1.47 [1.40, 1.54]	50.7 [50.3, 51.2]	1.89 [1.80, 1.98]	97.6 [97.5, 97.8]	1.91 [1.85, 1.97]
Other hospital	25.1 [24.4, 25.7]	1.13 [1.04, 1.22]	41.7 [40.9, 42.4]	1.08 [1.02, 1.15]	31.9 [31.2, 32.6]	0.99 [0.93, 1.06]	93.8 [93.5, 94.2]	1.57 [1.52, 1.63]
Clinic	21.3 [20.9, 21.7]	1 (ref)	39.6 [39.1, 40.1]	1 (ref)	31.2 [30.7, 31.7]	1 (ref)	88.9 [88.6, 89.3]	1 (ref)

Cumulative incidences and hazard ratios are shown with the 95% confidence intervals

The cumulative incidences of testing for the years 2018 through 2022 are presented in the rows corresponding to the respective year. The additional rows illustrate the cumulative incidence of testing within the overall population, stratified by factors such as drug type or insurance type

Hazard ratios were estimated by using the Cox regression model with a log-normal frailty term to account for unobserved heterogeneity across facilities. The model incorporated all covariates simultaneously

*CBC* complete blood count, *CIF* cumulative incidence function, *DPC* Diagnosis Procedure Combination, *ESAs* erythropoiesis-stimulating agents, *HIF-PH* hypoxia-inducible factor-prolyl hydroxylase, *HR* hazard ratio, *TIBC or UIBC* total iron binding capacity or unsaturated iron binding capacity

## Discussion

Our nationwide analysis found inadequate adherence to testing for iron indices before and after the first prescription of ESAs or HIF-PH inhibitors for anemia in patients with CKD. The prevalence of testing for iron indices increased gradually, especially after the publication of the Japanese Society of Nephrology’s recommendations for the appropriate use of HIF-PH inhibitors in 2020. Facility type was a strong determinant of adherence, with further substantial variation observed across facilities, as indicated by the median prevalence ratios and the median hazard ratios.

This study found that a substantial proportion of patients were not tested for iron indices, similar to previous studies [5, 25]. For example, the CKD Outcomes and Practice Patterns Study, an ongoing international prospective cohort study, reported that 40–61% of patients with hemoglobin levels < 12 g/dL underwent iron index testing within 3 months of the index hemoglobin measurement [5]. Even though all patients in our study were new users of ESAs or HIF-PH inhibitors, testing was not universally implemented, highlighting opportunities for improved adherence to the recommendation.

A systematic review targeting patients undergoing hemodialysis demonstrated that appropriate intravenous iron supplementation can substantially lower ESA dosing requirements [26]. Although data on patients not receiving dialysis or the effect of oral iron supplementation are lacking, this potential for cost reduction underscores the importance of conducting timely tests, especially when using ESAs or HIF-PH inhibitors, to ensure that adequate iron supplementation is not overlooked.

The gradual increase in the prevalence of testing for iron indices can be attributed to the Japanese Society of Nephrology’s recommendations on the appropriate use of HIF-PH inhibitors, which were published in 2020 and emphasized monitoring iron indices. While we did not assess the direct impact of this publication, we are unaware of any other factors that might explain this uptick in testing.

The differences in recommendation-adherent testing across types of healthcare facilities were notable. Possible explanations include variations in medical expertise and resource availability. University hospitals or larger facilities may benefit from nephrologists familiar with expert society recommendations, facilitating better adherence. Such hospitals may also have greater access to diagnostic test results, enabling more informed treatment decisions regarding ESAs and HIF-PH inhibitors. Addressing these disparities may require targeted educational interventions [27], earlier nephrology consultations, and closer collaboration between generalist and specialist physicians [28]. Implementing clinical decision support systems [29] with large language models [30] and automated ordering systems may also help overcome the current scarcity of testing. However, the lower adoption rates of electronic health records in smaller hospitals and clinics in Japan present a challenge [31, 32]. Incentivizing the adoption of electronic health records among smaller healthcare providers may be necessary to bridge this gap.

## Limitations

This study has some limitations. First, we were unable to assess the appropriateness of ESA or HIF-PH inhibitor prescriptions due to the unavailability of laboratory data such as hemoglobin levels. The current CKD guidelines in Japan recommend initiating treatment for anemia in CKD to prevent hemoglobin levels from falling below 10 g/dL while avoiding levels above 13 g/dL. Second, physician-level data—such as identifiers or distinctions between generalists and specialists—were unavailable. A physician-level analysis could provide further insights into care variability and adherence patterns. Third, while testing before the initiation of these medications could be a more relevant quality indicator, testing after initiation may depend on the clinical situation. For example, if the medication was administered only once and then discontinued, assessing the iron status within 90 days after starting the medication may not always be necessary.

## Conclusions

The type of facility was the primary determinant of adherence to the recommendation for testing iron indices before the initiation of ESA or HIF-PH inhibitor therapy. Targeted educational interventions in low-adherence settings may help improve adherence rates and optimize patient care.

## Supplementary Information

Below is the link to the electronic supplementary material.

Supplementary file1 (DOCX 18 KB)
