# Supplementary material for: Adherence to monitoring iron indices at the initiation of erythropoiesis-stimulating agents or hypoxia-inducible factor prolyl hydroxylase inhibitors
Source: Clin Exp Nephrol. 2025 Sep 16;30(1):57–64. doi: 10.1007/s10157-025-02761-1 (PMC12811317; doi:10.1007/s10157-025-02761-1)
Supplement: Supplementary file 1 — Supplementary file1 (DOCX 18 KB) [file 10157_2025_2761_MOESM1_ESM.docx]

Supplementary tables

Title: Adherence to monitoring iron indices at the initiation of erythropoiesis-stimulating agents or hypoxia-inducible factor prolyl hydroxylase inhibitors

Yoshihisa Miyamoto, Akira Okada, Yusuke Sasabuchi, Masaomi Nangaku, Hideo Yasunaga

Corresponding author

Yoshihisa Miyamoto, MD, PhD

Department of Real-world Evidence, The University of Tokyo

7-3-1 Hongo, Bunkyo-ku, Tokyo 113-0033, Japan

Tel: +81-3-3815-5411

Fax: +81-3-3818-3762

Email: ymiyamoto70@gmail.com, ymiyamoto70-tokyo@umin.ac.jp

Supplemental table 1. WHO ATC Code or Receipt Computer Processing Code (9-digit numerical code)

| **Drug category** | **WHO ATC Code or Receipt Computer Processing Code (9-digit numerical code)** |
| --- | --- |
| Erythropoietin | B03XA01 |
| Darbepoetin | B03XA02 |
| Methoxy polyethylene glycol-epoetin beta | B03XA03 |
| Enarodustat | 622829501,622829601 |
| Daprodustat | B03XA07 |
| Molidustat | 622854901,622855001,622855101,622855301 |
| Roxadustat | B03XA05 |
| Vadadustat | B03XA08 |
| Iron | B03AA,B03AB,B03AC |
| Anti-hyperglycemic drugs | A10 |
| Antihyperlipidemic drugs | C10 |
| Antihypertensive drugs | C02,C03,C07,C08,C09,C10BX03 |
| Vitamin D | A11CC |
| Iron binder | V03AE05,V03AE08 |
| Phosphate binder | A12AA04,A02AC01,V03AE03,V03AE02,  622516401,622149201 |

Supplemental table 2. ICD10 Codes

| **Diseases** | **ICD 10 codes** |
| --- | --- |
| Chronic kidney disease | N18 |
| Stroke | G450, G451, G458, G459, I269, I609, I619, I630, I631, I632, I633, I634, I635, I678, I74 |
| Congestive heart failure | I099, I110, I130, I132, I255, I425, I426, I427, I428, I429, I43, I50, P290 |
| Myocardial infarction | I21, I22, I252 |
| Myelodysplastic syndrome | D46 |

ICD: International Classification of Diseases

Supplemental table 3. Procedural codes

| **Procedures** | **Codes** |
| --- | --- |
| Autologous blood donation | 150327610, 150327710, 150327510 |
| Dialysis | 114003510, 114009310, 140059510, 140036710, 140060210, 140059310, 140060310, 140057810, 140051010, 140051110, 140060410, 140059410, 140057910, 140058010, 140008510, 140060610, 140058310, 140058110, 140060710, 140052810, 140060510, 140058210, 140008810, 140061010, 140060910, 140058510, 140058410, 140060810, 140058610 |
| Complete blood count or other hematological assessment | 160191510, 160061810, 160008010, 160008210 |
| Measurement of ferritin | 160192510, 160036810 |
| Measurement of serum iron | 160022110 |
| Measurement of total iron-binding capacity or unsaturated iron-binding capacity | 160023610, 160023710, 160028810, 160028710 |
